# Superior pattern processing is the essence of the evolved human brain

**DOI:** 10.3389/fnins.2014.00265

**Published:** 2014-08-22

**Authors:** Mark P. Mattson

**Affiliations:** ^1^Laboratory of Neurosciences, National Institute on Aging Intramural Research ProgramBaltimore, MD, USA; ^2^Department of Neuroscience, Johns Hopkins University School of MedicineBaltimore, MD, USA

**Keywords:** evolution, hippocampus, language disorders, religion and science, neuronal network

## Abstract

Humans have long pondered the nature of their mind/brain and, particularly why its capacities for reasoning, communication and abstract thought are far superior to other species, including closely related anthropoids. This article considers superior pattern processing (SPP) as the fundamental basis of most, if not all, unique features of the human brain including intelligence, language, imagination, invention, and the belief in imaginary entities such as ghosts and gods. SPP involves the electrochemical, neuronal network-based, encoding, integration, and transfer to other individuals of perceived or mentally-fabricated patterns. During human evolution, pattern processing capabilities became increasingly sophisticated as the result of expansion of the cerebral cortex, particularly the prefrontal cortex and regions involved in processing of images. Specific patterns, real or imagined, are reinforced by emotional experiences, indoctrination and even psychedelic drugs. Impaired or dysregulated SPP is fundamental to cognitive and psychiatric disorders. A broader understanding of SPP mechanisms, and their roles in normal and abnormal function of the human brain, may enable the development of interventions that reduce irrational decisions and destructive behaviors.

## Introduction

The fundamental function of the brains of all animals is to encode and integrate information acquired from the environment through sensory inputs, and then generate adaptive behavioral responses. Sensory information is first rapidly encoded as patterns inherent in the inputs, with visual and auditory patterns being most extensively studied in mammals (Wang et al., 2009; Sweeny et al., 2011). The large numbers of encoded images and sound patterns can then be recalled and mentally manipulated in ways that enable comparisons of different patterns and, at least in the human brain, the generation of new patterns that convey objects and processes that could possibly exist, or are impossible or implausible. In this article, I define pattern processing as the encoding and integration of perceived or mentally-fabricated patterns which can then be used for decision-making and for transfer of the patterns to other individuals. Examples of types of pattern processing that are common among non-human primates and, in many instances, lower mammals and are therefore not uniquely human include: (1) Cognitive maps of the physical environment, such as the encoding and recall of locations of food sources, potential predators and navigation landmarks; cognitive mapping is critically dependent upon the hippocampus (Pearce et al., 1998; Spiers et al., 2001); (2) The ability to distinguish individuals of the same species, and their emotional state, based on features of their faces (Little et al., 2011; Parr, 2011; Yovel and Freiwald, 2013); (3) The use of gestures to capture the attention of, and to communicate a desired response from, other individuals (Liebal et al., 2004). It has been suggested that communication via gestures, which is well-characterized in apes, was a precursor to language during human evolution (Liebal et al., 2004; Tomasello, 2008).

The cognitive repertoire of humans far exceeds that of all other animals, and understanding the neurobiological basis of this superiority is therefore of interest not only to scientists, but also to society. As humans evolved from their anthropoid ancestors, and the size of their cerebral cortex expanded, novel pattern processing capabilities emerged. The main purposes of the present article are to describe the superior pattern processing (SPP) capabilities of the human brain, to forward the hypothesis that SPP is the neurobiological foundation of human sociocultural evolution, and to describe the roles of aberrant SPP in some major neurological disorders. The types of pattern processing that appear to occur robustly, if not uniquely in the human brain and are therefore considered as SPP include: (1) Creativity and invention, which have resulted in the development of tools, processes and protocols for solving problems and saving time, and the arts (Goel, 2014; Orban and Caruana, 2014; Zaidel, 2014). Examples include all aspects of agriculture, transportation, science, commerce defense/security, and music; (2) Spoken and written languages that enable rapid communication of highly specific information about all aspects of the physical universe and human experiences; (3) Reasoning and rapid decision-making; (4) Imagination and mental time travel which enables the formulation and rehearsal of potential future scenarios; and (5) Magical thinking/fantasy, cognitive process that involves beliefs in entities and processes that defy accepted laws of causality including telepathy, spirits, and gods (Einstein and Menzies, 2004). A major purpose of the present article is to forward the proposal that not only is pattern processing necessary for higher brain functions of humans, but SPP is sufficient to explain many such higher brain functions including creativity, imagination, language, and magical thinking.

**Figure 1:**
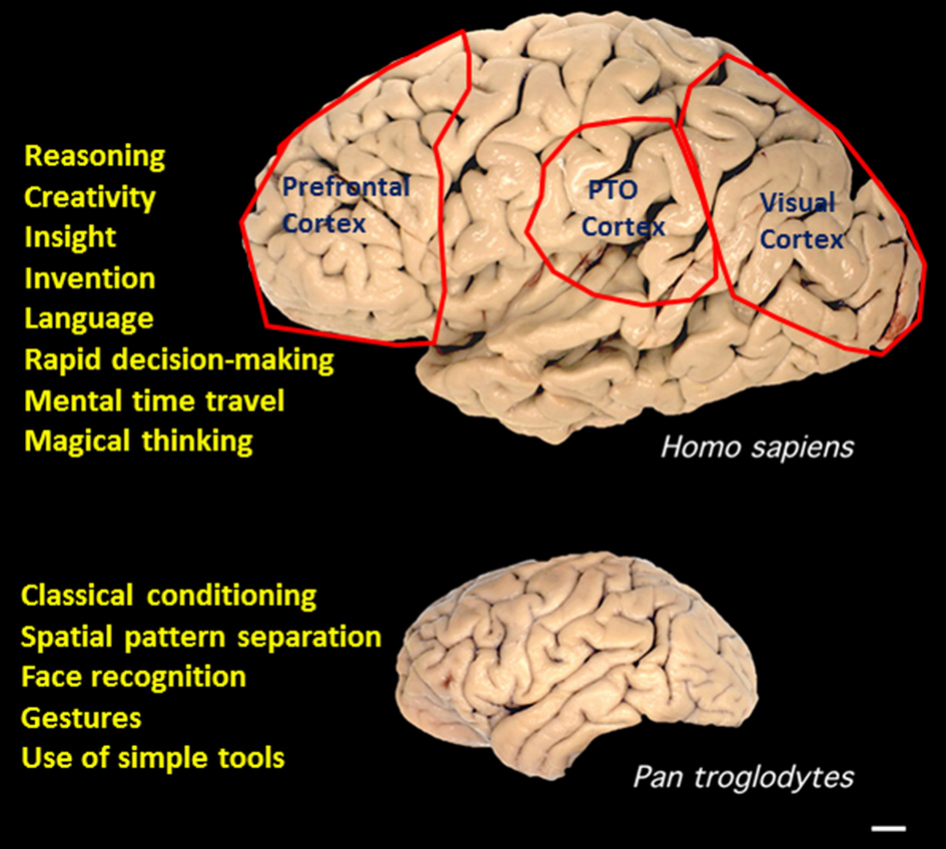
**Superior pattern processing (SPP) capabilities of the human brain evolved in association with expansion of the cerebral cortex**. A comparison of the gross anatomy of the brains of humans and chimpanzees (*Pan troglodytes*) reveals considerable expansion of three regions in humans, the prefrontal cortex, the visual cortex, and the parietal—temporal—occipital juncture (PTO). Examples of SPP capabilities of humans are listed next to the human brain. Examples of pattern processing capabilities of great apes are listed adjacent to the image of the chimpanzee brain. Source of brain images is Wikimedia Commons. Scale bar, 1 cm.

The results of functional brain imaging studies and of patients with brain injury suggest that multiple brain regions and neuronal networks are involved in each of the different types of SPP. Evolutionary considerations suggest that three brain regions may be particularly important in SPP in humans, the visual cortex, the prefrontal cortex and the parietal—occipital—temporal juncture (Figure 1). In humans, the cerebral cortex involved in processing visual inputs is considerably larger than lower anthropoids, likely due an evolutionary transition from being nocturnal, arboreal and relatively solitary, to being diurnal, ground-based and social (Passingham and Wise, 2012). Two major changes in the visual system selected for during this transition were forward-positioned eyes to allow binocular vision and depth perception, and changes in the structure of the retina to include a fovea and color vision (Kaas, 2012). The abilities to efficiently process complex visual and auditory patterns are also particularly important because of their centrality to language. Functional brain imaging studies have shown that, in addition to Broca's and Wernicke's regions (Damasio and Geschwind, 1984), language is processed in a distributed neuronal network that involves multiple cortical regions (Dick et al., 2013). While several regions of cerebral cortex are larger in the human brain compared to other anthropoids, the region that evolved most in the human lineage is the prefrontal cortex (Passingham and Wise, 2012). Some functions of the prefrontal cortex have been revealed by neuroanatomical, lesion and imaging studies and include insight and rapid decision-making (Passingham and Wise, 2012), episodic memory (Allen and Fortin, 2013), and complex social behaviors (Teffer and Semendeferi, 2012). Detailed consideration of the regional neuroanatomy of the different types of SPP is well beyond the scope of the present article, and is considered in recent articles as follows: creativity (Zaidel, 2014); language (Hagoort and Indefrey, 2014); imagination and mental time travel (Polyn and Sederberg, 2014) and magical thinking (Badzakova-Trajkov et al., 2011).

## Pattern processing is an evolutionarily conserved function of nervous systems

**“**The difference in mind between man and the higher animals, great as it is, is certainly one of degree and not of kind.” Charles Darwin, *The Descent of Man*.

What is the reason that the brain of our species is capable of cognitive feats well beyond even our closest non-human primate relatives? The human brain is remarkably similar to the brains of non-human primates and lower mammals at the molecular and cellular levels, suggesting that the human brain deploys evolutionarily generic signaling mechanisms to store and retrieve large amounts of information and, most remarkably, to integrate information in ways that result in the generation of new emergent properties such as complex languages, imagination, and invention. Here I review neurobiological aspects of pattern processing in birds and lower mammals and propose that an evolutionarily rapid expansion of pattern processing capabilities is the major reason that the human brain has capabilities considerably beyond those of lower species.

From homing pigeons to the swallows that return from Argentina to Capistrano each year, many bird species are capable of highly precise navigation. Accumulating evidence suggests that migrating birds encode navigational maps within the neuronal circuits of their brains. For example, in one study it was shown that adult crowned sparrows navigate toward a wintering area known from the previous year and can successfully correct for displacements of over 1000 miles (Thorup et al., 2007), suggesting the existence of a long-range experience-dependent global positioning system in the brains of these animals (Thorup and Holland, 2009). Acquiring food (foraging) and storing food are two spatial PP-mediated behaviors that, as in mammals (see next section) involve neural circuits in the hippocampus and associated higher cortical structures involved in sensory (particularly vision and olfaction) integration and processing (Bingman et al., 2003). The size of the hippocampus has been shown to change by as much as 30% in response to food-storing experiences and the increased hippocampal size associated with memorizing the location of food caches involves an increase in the total number of neurons in this brain region (Clayton, 1998; Biegler et al., 2001). Song learning and imprinting are two other well-known and well-studied types of learning and memory that are, as with all forms of higher learning, based on pattern processing (Healy and Hurly, 2004). As is true for mammals, learning and memory processes involved in pattern processing in birds involve activity at synapses that deploy the neurotransmitters glutamate, GABA and norepinephrine (Gibbs et al., 2008).

The human brain has retained many features of brain structure and cellular organization of the brains of birds and lower mammals, but has greatly elaborated upon them by developing more robust cortical neuronal networks involved in the processing of visual and auditory patterns. As in lower mammals, being aware of one's position in the environment, and remembering the locations of resources (food, shelter, etc.) and hazards (predators, cliffs, etc.) is of fundamental importance for the survival of humans. However, the encoding of visual inputs into “cognitive maps” of spatial relationships between objects in the environment (spatial pattern separation), and the encoding of auditory inputs, is necessary but not sufficient for the advanced PP abilities of humans including imagination, invention, and pattern transfer (language). The evidence suggests that expansion of the visual cortex, prefrontal cortex, and parietal—temporal—occipital (PTO) association area enabled the SPP that defines the human intellect capacity and all of its manifestations, including consciousness, language and mental fabrication and time travel. The remainder of this article describes some of the salient evidence for SPP as the basis of most, if not all, higher cortical functions in humans.

## Neural substrates of superior pattern processing: a conserved dynamic cytoarchitecture of intellect

The processing of visual images sensed by the eyes involves transfer of the features of those images to neuronal circuits in the primary visual cortex where the images are encoded in neurons in spatially-localized and oriented receptive fields comprised of cooperating neural networks that encode object identity and location (Rao and Ballard, 1998). The well-known homunculus of the primary somatosensory cortex provides a clear neuronal network structure-based map of the physical location on the body of the sensory receptors for pressure, pain, and temperature. Pattern encoding by the visual and somatosensory systems is therefore relatively easy to understand because the location of the sensory receptors themselves is “imprinted” in the cellular architecture of the corresponding primary sensory cortices. On the other hand, pattern encoding of auditory input is based largely on the quality and temporal ordering of sounds. In the cases of taste and olfaction, the quality of the tastes and odors is critical information that is encoded, but at the level of the neuronal networks and synapses pattern encoding occurs by mechanisms similar to those of the auditory system. Thus, odors evoke complex spatiotemporal activation of olfactory bulb neurons and a firing rate-based representation of the odors in the piriform cortex (Haddad et al., 2013).

While bigger in size, when viewed under the microscope the human brain is remarkably similar to those of non-human primates and lower mammals. At the cellular and molecular levels, there is little to distinguish our brain from those of species that preceded us in evolutionary history. The major cell types (neurons, astrocytes, oligodendrocytes, and microglia) are similar in their morphological features, molecular phenotypes and functions. Neurotransmitters are identical with glutamate and GABA being the major excitatory and inhibitory transmitters, and monoamines and acetylcholine being prominent secondary transmitters in all mammals. Similarly, gasotransmitters (nitric oxide, carbon monoxide, and hydrogen sulfide), neuropeptides and neurotrophic factors (e.g., neurotrophins, fibroblast growth factors, and insulin-like growth factors) are conserved (Amaral and Campbell, 1986; Jones, 1986; Barde, 1994; Mustafa et al., 2009). In addition, a range of behaviors including learning and memory, anxiety, aggression and others are modified in the same ways by the same drugs in humans and lower mammals. Moreover, genes that encode proteins involved in brain development, function and/or disease are largely conserved; indeed, genetic mutations that cause psychiatric or neurodegenerative disorders in humans often induce similar neurochemical, cellular and behavioral phenotypes in transgenic animals (McGonigle, 2013). Thus, findings from neuroscience research has confirmed the general conclusion of Charles Darwin who proposed in *The Descent of Man* that the minds of humans and related species are fundamentally similar (Darwin, 1871).

While some principles by which the brain uses pattern recognition and encoding to represent the past and the future have been established, a clear understanding of the underlying molecular and cellular mechanisms is lacking. How the encoded patterns are recalled and processed to generate enduring memories of the different patterns and their association with other encoded patterns (e.g., associations of the image of an object with the sound, smell, or feel of that object) is also not well understood. Nevertheless, the human brain is capable of using stored information to generate novel images, sounds, and other patterns in the processes of imagination and invention.

**Figure 2:**
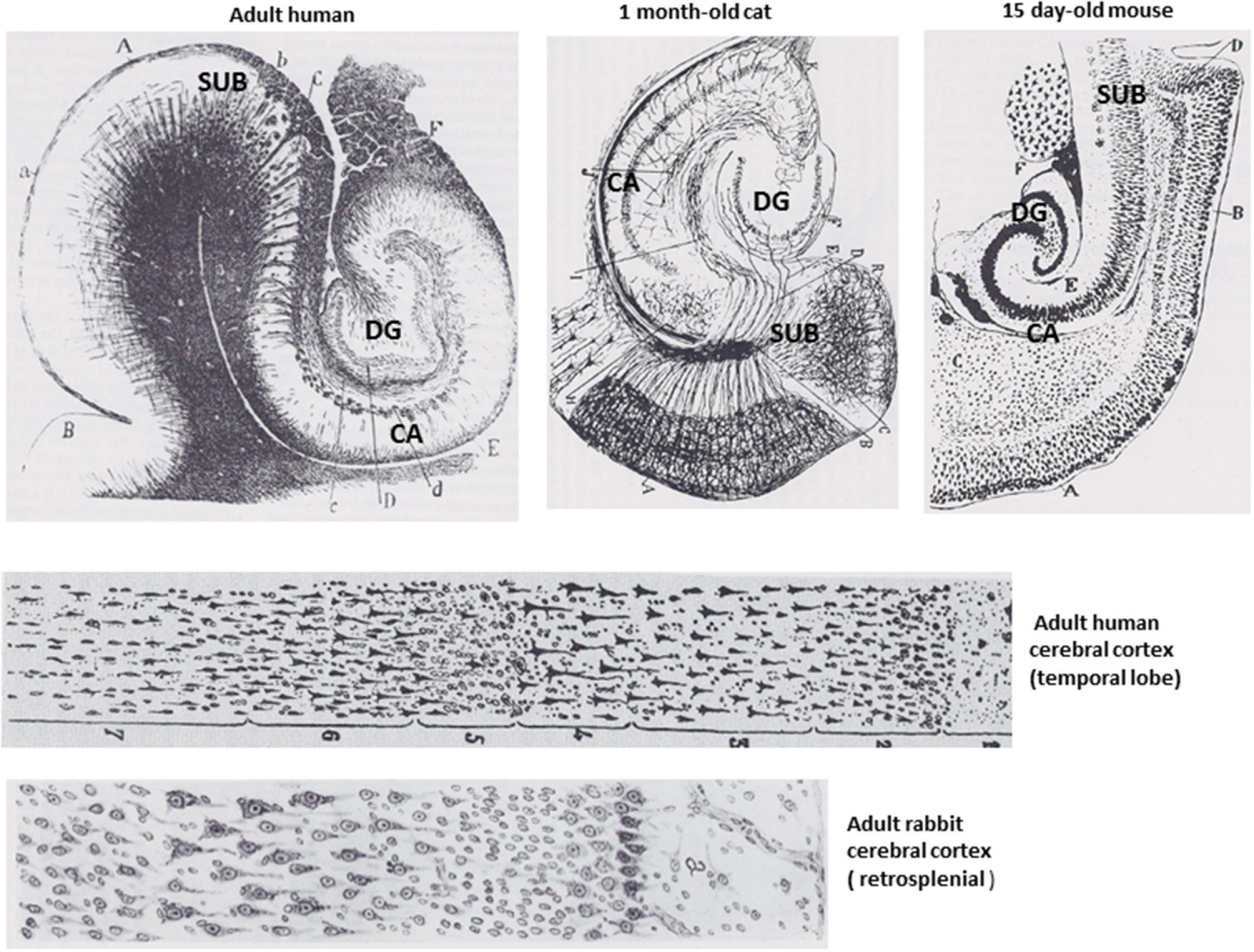
**Structural features of the brains of mammals are conserved from rodents to humans**. The upper drawings show the hippocampal formation of an adult human, a kitten and a young mouse. The lower two drawings show the cellular organization of the cerebral cortex of an adult human and an adult mouse, both of which exhibit six cell layers. All of the drawings are adapted from Santiago Ramon y Cajal (DeFelipe and Jones, 1988). CA, cornu ammonis; DG, dentate gyrus; SUB, subiculum.

The term intelligence has been defined in different ways by psychologists and neuroscientists, but a general definition proposed by one group of eminent scholars is “A very general mental capability that, among other things, involves the ability to reason, plan, solve problems, think abstractly, comprehend complex ideas, learn quickly, and learn from experience.” (Gottfredson, 1997). All mammals are able to learn and to make decisions and choices based upon their prior learning experiences, a fundamental aspect of reasoning. Mice and rats have about 1 billion neurons in their nervous system whereas humans have 100-fold more neurons with approximately 30 billion being in the brain. Neuroanatomical and neurochemical considerations described in this section suggest that the superior intellectual capabilities of humans are solely or largely the result of the increase in the number of neurons and synapses that mediate enhanced encoding, integration and inter-individual transfer of patterns. As referenced above, there is little or no uniqueness in the structural or functional properties of the neuronal circuits that mediate intelligence in humans (Figure 2). Moreover, the intellectual capability of any individual requires the integrated function of pattern-processing networks distributed throughout the cerebral cortex (Duncan, 2010), indicating that there is no single brain structure responsible for the mental superiority of humans.

The cellular and molecular mechanisms of pattern processing have been most intensively investigated in nerve cell circuits of the hippocampus, a brain region that plays a critical role in spatial learning and memory (Yassa and Stark, 2011). When our eyes and ears are open they are continually detecting images and sounds, the details of which are transiently encoded in nerve cell circuits in the visual and auditory regions of the cerebral cortex, respectively. The neurally-encoded visual and auditory patterns (as well as olfactory, gustatory and touch/pain inputs) are then transferred into hippocampal circuits via neurons in the entorhinal cortex. Inputs from the other senses (olfactory, gustatory, touch/pain) are also communicated to the hippocampus. Learning and long-term memories of such experiences require the hippocampus in humans, monkeys, rodents, and birds (Stella et al., 2012). For example, birds that store food in caches are able to remember, in a hippocampus-dependent manner, not only the locations of the caches, but also what they placed in the cache and when they put it there. Moreover, such birds can modify their caching strategy when they perceive that another bird (a potential pilferer) is watching them (Emery and Clayton, 2001). Thus, similar to humans, these birds have an episodic memory system that is modified by social context.

Electrical recordings from neurons in the hippocampus of freely-moving rats have shown the existence of “place cells,” neurons that fire action potentials when the rat is in a specific location of the same test arena (Moser et al., 2008; Burgess and O'Keefe, 2011). Other neurons fire in response to cues within the test chamber, regardless of the location of the test arena, suggesting that hippocampal circuits encode both spatial and episodic memory information (Leutgeb et al., 2005). Lesions of neurons in regions CA1 and CA3 of the hippocampus in rats result in impaired ability to remember a sequence of spatial locations (Lee et al., 2005), and selective destruction of dentate granule neurons results in an impaired ability of rats to learn adjacent spatial locations in a radial arm maze (Morris et al., 2012). Remarkably, a recent study demonstrated the neuronal network activity basis of the envisioning of the location of a food source by rats (Pfeiffer and Foster, 2013). Electrical activity was simultaneously recorded from approximately 200 neurons in the hippocampus of rats while they were foraging for food. Prior to initiating navigation toward a potential food source, there occurred activity in a sequence of neurons (place cells) that accurately tracked the progression of the rat from its current location to the remembered location of the food source. These findings provide evidence that rats are capable of visual imagery in that neuronal ensembles that encode a specific location are activated when the rat is “contemplating” that location.

Neurons in the dentate gyrus of the hippocampus play a particularly important role in spatial pattern separation (PS) in mammals (Gilbert et al., 1998). Interestingly, and in contrast to other brain regions, there are neural stem cells in the dentate gyrus that are capable of differentiating into granule neurons that then integrate into the existing hippocampal circuitry. Animal studies have shown that spatial PS is impaired by selective destruction of hippocampal stem cells (Clelland et al., 2009) while spatial pattern processing is enhanced by manipulations such as exercise that stimulate neurogenesis (Creer et al., 2010; Sahay et al., 2011). The newly generated neurons receive sequential inputs from other granule neurons (transient), CA3 pyramidal neurons, cholinergic neurons in the basal forebrain and glutamatergic neurons in the entorhinal cortex (Vivar et al., 2012; Vivar and van Praag, 2013). Inputs from GABAergic interneurons increase gradually over a period of several months. Young granule neurons are critical for PS, whereas older granule neurons facilitate pattern completion (Nakashiba et al., 2012). Interestingly, spatial PS can be enhanced by exercise (running) in mice (Creer et al., 2010), and young adult humans perform better than older adults in spatial PS tests (Holden et al., 2012). As described in the next two paragraphs, the enhancement of PP by running likely played an adaptive role during human evolution, with the hypothesis being that individuals with superior hippocampus-dependent spatial pattern separation, which involves neurogenesis, would have a survival advantage because of the presumed importance of spatial pattern separation in remembering the location of objects in space and time (food sources, predators, etc.).

One prominent phenotypic change that is believed to have occurred during the evolutionary transition from the Genus *Pan* (chimpanzees) to the Genus *Homo* (approximately 5–8 million years ago), was the acquisition of an upright bipedal endurance/distance runner phenotype (Bramble and Lieberman, 2004; Lieberman and Bramble, 2007; Mattson, 2012). Bipedalism also enabled the evolution of the shoulder in ways that allowed humans to throw objects accurately at a high velocity, greatly improving their ability as hunters (Roach et al., 2013). This was also the period in the evolution of our species when the size of the cerebral cortex increased relatively rapidly, which suggests that the expansion of the territory covered by individuals and groups of humans (enabled by endurance running) played a role in the expansion of the cerebral cortex. Coverage of a larger territory during the great human expansion (Henn et al., 2012) would have provided the opportunity to access more resources (food, water, and shelter), and required a greater pattern processing capacity to remember details of the location and nature of the resources. Importantly, humans evolved the ability to transfer the information acquired and processed in their brains during their journeys to other individuals via gestures, map drawing, and language. Visual and auditory patterns were likely the most commonly processed and transferred because of the ability to readily and accurately reproduce sights and sounds. Accordingly, the regions of the brain that expanded in humans are mostly involved in pattern processing of sights and sounds, and their codification as written and spoken languages. Very interestingly, specialized motor training (sports) enhances language understanding by a mechanism involving recruitment of the left dorsal lateral premotor cortex, suggesting that the language system is functionally connected to motor skill-related areas outside of the core language networks (Beilock et al., 2008). The latter findings suggest that the language SPP capabilities of the human brain co-evolved with development of organized “teamwork,” which may have bolstered functional interactions between brain regions involved in language and those responsible for specialized sensory-motor skills.

How might endurance running have contributed to the SPP capabilities of humans? Among mammalian species there are significant positive correlations between brain size, cognitive abilities, and exercise capacity (Raichlen and Gordon, 2011). Studies of rodents, monkeys, and humans have shown that running can increase the size of several different brain regions including the hippocampus and midbrain (Rhyu et al., 2010; Erickson et al., 2011; Biedermann et al., 2012; Kolb et al., 2013). Presumably, individuals whose brains responded to endurance exercise by increasing the growth of their brain cells would have a survival advantage because of the superior PP ability conferred by the additional neural circuits. Indeed, at the cellular level running can increase numbers of synapses and the production of new neurons from progenitor cells in the hippocampus (Stranahan et al., 2009; van Praag, 2009). The ability of running to improve pattern processing is evolutionarily conserved, as demonstrated in experiments with rats and mice showing that running enhances hippocampus-dependent spatial pattern separation (Griffin et al., 2009; Creer et al., 2010). In humans, running improves mood and enhances cognitive and sensory—motor capabilities (Nabkasorn et al., 2006; Winter et al., 2007; Stroth et al., 2010), and running also enhances cognitive performance in monkeys (Rhyu et al., 2010). Interestingly, intermittent fasting (which simulates the natural condition of limited food availability encountered by our ancestors) can also enhance cognitive function and increase neuronal network complexity (Martin et al., 2008; Singh et al., 2012; Li et al., 2013). The latter findings are consistent with the possibility that individuals best able to enhance their cognitive function when faced with the challenge of food scarcity are most likely to find food and survive. The neurochemical processes that may link endurance running and fasting/hunger to heightened cognitive performance include the neurotransmitters glutamate, serotonin and norepinephrine and the neurotrophic factor brain-derived neurotrophic factor (BDNF), all of which are critically involved in the neuronal network plasticity required for pattern processing (Richter-Levin et al., 1995; Barkus et al., 2010; Rothman et al., 2012).

Intelligence involves not only the ability to encode large amounts of detailed pattern information, but also the ability to integrate that information and make appropriate (adaptive) decisions. Nerve cell circuits in the frontal cortex play a major role in decision-making in humans and lower mammals as well. Studies of patients who underwent neurosurgical removal of their hippocampus or frontal lobe on one side of their brain demonstrate that the neural circuits in these two brain regions mediate complementary aspects of SPP, namely, visual working memory (hippocampus), and programs of complex behaviors, decision-making and planning for the future (frontal cortex) (Owen et al., 1996; Barbey et al., 2009). Animal studies support the critical importance of the hippocampus, but not the medial prefrontal cortex (mPFC), in spatial pattern separation and further show the importance of both brain regions in working memory (McAllister et al., 2013). Because spatial pattern separation is a type of pattern processing that is conserved among all mammals, it is not considered SPP. However, while not sufficient to explain SPP capabilities of humans, spatial pattern separation is fundamental to the initial encoding of the patterns (letters, words, and other visual images) that are necessary for SPP (language, invention; imagination, mental time travel).

Iriki et al. (see Iriki and Sakura, 2008 for review) performed a fascinating set of studies in which they trained Japanese macaques to retrieve food located beyond their reach using a rake. This species of monkey does not use tools in the wild. Because evidence from human studies suggest that such body image-related tool using skills involve strengthening of synaptic connectivity in the parietal cortex, Iriki et al. performed electrophysiological recordings and positron emission tomography imaging studies of this brain region. They found that learning to use a rake was associated with an expansion of the functional representation (receptive fields) in the parietal cortex of the hand used to hold the rake. Using tract tracing methods, the authors found increased growth of axons and synaptogenesis in the intraparietal cortex of monkeys trained to use the rake tool compared to untrained monkeys. Based upon their findings in monkeys and studies of intellect in humans, the authors propose (Iriki and Sakura, 2008) that a major advance in human evolution was the process of “intentional niche construction” in which patterns encoded from prior experiences are used to construct new patterns that allow for prediction of the consequences of future actions within new niches/situations. This suggests that the mental manipulation of patterns to “rehearse” future scenarios is a capability of non-human primates, albeit dramatically less advanced than humans.

Emerging evidence, recently reviewed by Hunsaker and Kesner (2013), reveals that SPP occurs not only in the cerebral cortex, but also in other brain regions. Structures such as the striatum and cerebellum which are best known for their roles in the control motor function are also involved in episodic learning and memory, and decision-making processes. Neurons in the dorsal striatum are involved in procedural memory and also communicate with neurons in the ventral striatum which, in turn, integrate inputs from the hippocampus and prefrontal cortex to generate goal-directed behaviors (Pennartz et al., 2011). The cerebellum is critical for motor learning which involves the same kinds of synaptic modifications (e.g., glutamate receptor-mediated LTP and LTD) that underlie spatial learning encoded by hippocampal neurons (Gao et al., 2012). The learning of complex skills, such as those required to become adept at a sport, music or craft, involves neuronal networks that span a broad array of CNS structures (Schlaug, 2001). For example, fMRI was used to evaluate regional activities in the brains of shooters before and after 90 h of shooting training (Baeck et al., 2012). During mental imagery of shooting prior to or after shooting practice, the subjects exhibited activity in widely distributed regions of the cerebral cortex. However, the basal ganglia exhibited a distinct increase in activity only after shooting practice, suggesting that perfecting a shooting skill involves more focused neural correlates that may involve formation of new synapses. Electroencephalogram recordings of event-related brain potentials in adolescents with different amounts of musical training provided evidence that training improves the ability to detect sound patterns across longer time intervals (Wang et al., 2009). The latter findings are an example of experience-dependent expansion of pattern processing capability.

Functional modularity and a hierarchical “command and control” system provide important evolutionary frameworks for how the human brain works (Geary and Huffman, 2002; Barrett, 2012). The modularity model (Geary and Huffman, 2002) would predict that SPP functions of the human brain involve functional modules of neuronal circuits that include multiple brain regions working together to achieve their task (rapid decision making, insight, imagination, language, etc.). Studies of the connectivity of the prefrontal cortex with other brain regions provides strong evidence for such functional modularity (Passingham and Wise, 2012). Evolutionary considerations suggest that many neuronal circuits in the human brain are hierarchically organized with certain “design” features shared among different brain regions, while other design features are specific for, or tuned to, specific higher cortical functions (Barrett, 2012). The control of such trans-regional neural circuits likely involves a hierarchical mechanism in which a particular neuronal network plays a dominant, but not exclusive, role in a particular type of SPP.

## Emotions reinforce pattern processing

“We have seen that the senses and intuitions, the various emotions and faculties, such as love, memory, attention, curiosity, imitation, reason, etc., of which man boasts, may be found in an incipient, or even sometimes well-developed condition, in the lower animals. “Charles Darwin, *The Descent of Man*.”

Emotions such as fear, anger, pleasure, and love are elevated states of arousal that enhance memory and recall of the events occurring during those emotional states (Bergado et al., 2011; Maren et al., 2013). This is a major, if not singular, function of emotions. Emotions evolved to reinforce memories of patterns of particular significance vis-à-vis survival and reproduction. Remembering the details of the events of an attack by a predator or intra-species rival will increase the probability of avoidance of such potentially deadly encounters in the future. Memories of the pleasurable experience of intercourse with fertile individuals of the opposite sex provides motivation for additional bouts of intercourse, and so increases the probability of passing one's genes on to future generations. Pattern processing in its most fundamental manifestation is enhanced by perception of the patterns in an emotional setting. Thus, hippocampus-dependent spatial pattern separation is enhanced when human subjects are shown fearful stimuli prior to testing pattern separation (Segal et al., 2012). Studies of animal models and human subjects have revealed core mechanisms by which the memory of an emotional experience is strengthened. The neural circuits and neurochemical substrates of emotion-related cognitive processing are conserved among mammals, and include brainstem, limbic and cortical networks, as well as neuroendocrine signaling. Brainstem noradrenergic neurons, limbic and cortical glutamatergic neurons, and neuropeptidergic neurons such as those producing corticotropin-releasing hormone (CRH) and oxytocin play prominent roles in emotion-related cognitive enhancement (van Stegeren, 2008; Thoeringer et al., 2012). Epinephrine and cortisol, adrenal hormones produced in response to activation of brain fear and anger pathways, also enhance memories of the patterns perceived during the emotional experience (Fitzsimons et al., 2013; Toth et al., 2013).

Humans have evolved as highly social animals (Chang et al., 2013) with close emotional ties to mates, offspring, parents and close friends that enhance their survival and reproductive success (Damasio and Carvalho, 2013). As with other emotions, those associated with social interactions may have evolved to enhance SPP. In this view, there is a self-amplifying reciprocal relationship between social interactions and SPP ability. Thus, advanced PP abilities enable the development of social bonds and networks and, conversely, social interactions stimulate SPP. Success in social interactions requires that one recognize others, remember their past experiences with those individuals, and communicate their intentions. Dunbar's social brain hypothesis of evolution of the primate brain includes the possible role of emotional attachments to mates and friends in complex social networks in the expansion of the cerebral cortex during anthropoid evolution (Dunbar, 2009; Sutcliffe et al., 2012). Because the memories of specific patterns (faces, places, conversations, etc.) can be reinforced or even embellished by emotions (Holland and Kensinger, 2010), it is reasonable to consider that evolution of the social brain was bolstered by emotional relationships. In addition to their use of complex language (see next Section), humans have added another dimension to social interactions—they are aware that others have thoughts and emotions very similar to their own. Humans therefore not only encode and process patterns representing their own experiences, but also the experiences of their family, friends and workmates. Social interactions require processing of information regarding the histories, behaviors and thoughts of many other individuals. Whether family members, employees or competitors, there are clear advantages to being able to know what others have done in the past, and to predict their future behaviors. Thus, inter-personal SPP is critical for success in most aspects of life, including acquiring and retaining friends, a job and a mate. Emotions reinforce inter-personal SPP, such that interactions involving anger, pleasure, sadness, etc. are retained, recalled and processed more thoroughly than interactions occurring in a neutral emotional context.

## Languages as an advanced pattern encoding and transfer mechanism

Language is the quintessential example of the evolved SPP capabilities of the human brain as it involves (once learned) the instantaneous conversion of sounds to visual symbols, and vice-versa. Language is a complex behavior in which auditory and/or visual patterns learned from other individuals or perceived in the environment are encoded, processed and modified for the purpose of transfer of information to other individuals. Language involves the use of patterns (symbols, words, and sounds) to code for objects and events encountered either via direct experience or communication from other individuals. Language-related SPP can create new patterns (stories, paintings, songs, etc.) of “things” that may (reality) or may not (fiction) exist. Language-mediated encoding and transfer of auditory and visual patterns enabled the rapid evolution of the human brain and is likely a major reason for the current dominance of *Homo sapiens*. (Aboitiz et al., 2006; Berwick et al., 2013). Individuals with the ability to communicate to their family/tribe members the precise locations of food sources, hazards and other salient features of their environment would have had a clear survival advantage. In modern times, rapid advances in science, technology and medicine are facilitated by language-based information transfer. However, despite it being a remarkable leap forward in evolution, language may not involve any fundamentally new cellular or molecular mechanisms; instead, language is mediated by recently evolved neural circuits integrated with older circuits, all of which utilize generic pattern processing mechanisms. In this section I briefly summarize what is known of the evolutionary history of language, and then consider the underlying neural circuits and their highly efficient ability to detect, encode, manipulate, and then transfer patterns.

While birds and non-human primates exhibit auditory communication, their vocalizations convey general information such as danger, rather than detailed instructions. It has been proposed by Tomasello (2008) that the kinds of gestures used by great apes is an evolutionary precursor of language. Studies of infant humans further support the notion that pointing and gestures are an ontogenic precursor to language (Goldin-Meadow, 2007; Liszkowski et al., 2009). Languages involving complex vocabularies and written symbols and words are believed to have arisen in *Homo sapiens* beginning approximately 100,000 years ago (Berwick et al., 2013). The rapid evolution of language skills, and the underlying neural circuits that mediate language processes, is fully consistent with its fundamental role in the rapid advancement of human societies. Language provides powerful reproductive and survival advantages. A man who engages a woman in stimulating conversation is more likely to attract her as a mate than is an inarticulate man. An army whose soldiers use detailed maps and advanced communication skills is more likely to win a battle than is an army that charges forward “blindly.”

Language involves sensory and motor pathways, and associated cerebral cortical regions that encode and integrate the learned patterns of sounds and symbols/letters (Kuhl, 2010). Brain regions that are critically involved in language SPP in humans include ventrolateral prefrontal cortex and parietal cortex (Broca's area), and the posterior region of the superior temporal gyrus and adjacent parietal lobe (Wernicke's area) situated between the auditory and visual cortices. Broca's area is closely associated with motor cortex and, accordingly, is critical for the production of speech. Wernicke's area is critical for the comprehension of language and accurate communication via speech or writing. A comparison of human and macaque brains suggests there are two major “language pathways,” one that connects anterior auditory regions with ventrolateral prefrontal areas and one that connects auditory areas with ventrolateral prefrontal and parietal areas (Aboitiz, 2012). The second pathway is more highly developed for phonological and complex syntax processing, whereas in macaques it is involved more in hand and body gestures and involuntary vocalizations. Therefore, the elaborate vocal communication PP capability of humans has an evolutionary overlap with brain structures and circuits used for gesturing in non-human primates (Aboitiz, 2012). The neuronal circuits and mechanisms that evolved to enable communication using gestures are not well understood, but may involve a mirror system that matches observed events with similar intrinsically produced movements to enable communication of specific requests or intentions to another attentive individual (Rizzolatti and Arbib, 1998).

As they exist today, modern languages are the result of contributions of many generations of people living in the same territory/country. Once the basic set of symbols (letters) were established, specific patterns of the letters (words) were assigned to specific objects or phenomena, and the words were arranged in sequences to describe the temporal and/or physical relationships between the objects and phenomena. The number of words in the English language in common use (in a dictionary) is well over 200,000, but when one considers the number of discipline-specific words (in science, technology, engineering, etc.) the number of words is perhaps at least an order of magnitude greater. Moreover, for all intents and purposes there are an infinite number of sentences and stories that can be fabricated using even a small fraction of the total number of words. Remarkably, the learning of languages and the potentially infinite number of stories (word sequences) that an individual can construct are accomplished using a finite number of neurons that is established during early brain development. Our current understanding of the mechanisms of language learning, memory and communication is that they involve chemical and structural changes in synapses in neuronal circuits involved in encoding (hippocampus) and consolidation (multiple cortical regions) of words and the entities they represent (Shtyrov, 2012). Presumably, the synapses involved in language are “strengthened” by repetition (listening and talking, and reading and writing).

## Drawings and maps

“The soul never thinks without a picture”—Aristotle

Drawings, including maps, date to at least 30,000 years ago (Valladas et al., 2001; Pike et al., 2012). Language and drawings serve complementary purposes. Language provided humans with a novel behavioral tool that strengthened a pre-existing ability to integrate both geometric and non-geometric information. Drawing of objects while they are being observed requires alternating observation of the object and drawing, a process with ongoing rapid feedback that allows (with practice) fairly accurate rendering of the objects' features. Drawings of real objects recalled from long-term memory are less accurate in detail but can provide key aspects of the object which the drawer wishes to convey to viewers of the drawing.

From an evolutionary perspective, maps may have been particularly important. According to Landau and Lakusta (2009): “Maps are the symbolic system par excellence for encoding and permanently retaining spatial information. Unlike language, the format of maps is roughly analog in nature, capturing the spatial layout of an environment by spatial transformations that preserve what is essential to finding things—most often, the geometric properties of layouts.” Maps are particularly valuable because they include large amounts of information about spatial relationships that cannot be readily conveyed via language. Cognitive maps of our environs are routinely constructed, stored, and refined. The maps can then be drawn and used by others to locate places they have never previously been. Grid and place cells in the hippocampus and grid cells in the entorhinal cortex are believed to play key roles in generating cognitive maps by the process of path integration. The parietal cortex and presubiculum, and probably additional connected brain regions, are also likely involved in the generation of cognitive maps. Because the precision of drawings based on cognitive maps is limited, particularly with regards to distances between objects/landmarks, humans have developed technologies to generate accurate maps drawn to scale. Indeed, the measurement and recording of distances have advanced to include modern day laser range finders and satellite-based global positioning systems.

## Fabricated patterns: imagination, invention and magical thinking

“The same high mental faculties which first led man to believe in unseen spiritual agencies, then in fetishism, polytheism, and ultimately in monotheism, would infallibly lead him, as long as his reasoning powers remained poorly developed, to various strange superstitions and customs.… Yet it is well occasionally to reflect on these superstitions, for they show us what an infinite debt of gratitude we owe to the improvement of our reason, to science, and to our accumulated knowledge.” Charles Darwin, *The Descent of Man*.

While the ability to reproduce perceived patterns in drawings and maps is robust in humans, so too is the ability to create new patterns of entities that may or may not exist in the real world. To paraphrase Tattersall (2010) “the human brain has a superior ability to mentally manipulate animate and inanimate patterns into a myriad of intangible symbols that can then be recombined to produce new images of the world; we therefore live partly in worlds of our own mental creation, superimposed upon or distinct from the natural world.” Studies of the rapid eye movement (REM) (dreaming) state of sleep, and of various states of consciousness (see next two paragraphs), have provided insight into the neural networks involved in SPP that occurs in the brain in the absence of significant sensory inputs.

Functional MRI analyses of normal subjects in various states of consciousness (fully alert and engaged in behaviors, awake and resting, sleep states) and in anesthetized and brain-injured subjects have provided insight into where and how the brain processes patterns. When a healthy person lies quietly with their eyes closed for several minutes (the resting state) there is a network of neuronal circuits that are very active. This so-called default mode network (DMN) includes the mPFC, posterior cingulate cortex/precuneus (PCC), anterior cingulate cortex and parieto-temporal junction (Raichle and Snyder, 2007). A range of PP roles have been proposed for the DMN including introspection, social cognition, stimulus-independent thought, and integration of patterns/cognitive processes (Heine et al., 2012). For example, functional brain imaging studies suggest that the SPP capability of creativity is associated with strong functional coupling between the mPFC and PCC, two key nodes in the DMN (Takeuchi et al., 2012). The DMN activity and intra-DMN connectivity varies with consciousness state. For example, DMN activity in the PCC/precuneus is absent in brain dead patients, reduced in minimally conscious patients and further reduced in patients in a vegetative state (Vanhaudenhuyse et al., 2010). As subjects transit from the awake state to deep sleep there is reduced activity in the frontal DMN and reduced connectivity with posterior regions of the DMN (Horovitz et al., 2009). Patients in a minimally conscious state exhibit reduced connectivity within the DMN and between the precuneus and thalamus, and as the emerge from this state these connectivities increase (Fernández-Espejo et al., 2012).

The nature and function of sleep states has been a topic of considerable interest for centuries, with recent findings suggesting important adaptive functions that enhance brain function during wakefulness, most notably memory consolidation (Walker and Stickgold, 2004). Neural networks throughout the cerebral cortex are remarkably active during sleep (for review see Picchioni et al., 2013). In the REM stage of sleep there is increased activity in the hippocampus, cingulate and sensory cortices, and amygdala which may play roles in memory consolidation and regulation of emotions. Indeed, recall of dreams suggests that the PP occurring during REM sleep involves the mental construction of scenes that are often emotional in nature (e.g., a dangerous or disturbing situation). Based on recall of dreams, and emerging evidence regarding the neuronal circuits involved in dreaming, the types of SPP that occur during REM sleep in humans include language, imagination and magical thinking (De Gennaro et al., 2012). In non-REM light sleep there is a relative preservation of cortical network activities, while in deep (slow wave) sleep there is a marked decrease in activity of cortical networks. The DMN may play important roles in sleep states. During the transition to sleep activity among nodes within the DMN remains high, but interactions of the DMN with anti-correlated cortical networks is reduced. In deep sleep connectivity between the frontal and posterior nodes of the DMN is reduced. Until recently, studies of the DMN have been restricted to humans. However, a DMN that includes homologous brain regions has recently been described in rodents (Lu et al., 2012) which provides new opportunities to better understand the molecular and cellular workings of the DMN, and how dysfunction of the DMN contributes to aberrant PP and associated neurological disorders.

The importance of imagination and invention for the rapid advancement of the human species cannot be overstated. The invention of tools and technologies have dominated the recent development of civilizations throughout the world. The earliest evidence for the invention of tools by our human ancestors dates to approximately 2.5 million years ago in Ethiopia and Kenya where stones were fashioned into cutting tools (Plummer, 2004). At that time hominid brains were about the same size as those of apes (approximately 500 grams), whereas the brain of modern humans is nearly three times larger. Functional MRI studies suggest that when someone views and then imagines using a tool, widespread cortical neuronal networks are activated including those in the motor areas and temporoparietal, inferior frontal, occipital, parietal, and ventral temporal areas (Wadsworth and Kana, 2011). The latter findings are consistent with a scenario in which the ability to invent novel objects, technologies and computations likely required the expansion of circuits involved in the processing of patterns (particularly visual and auditory patterns) perceived in the environment or learned from others. Brain regions involved in episodic (chronologically ordered) memory of past events are also involved in the imagining of future events (Suddendorf et al., 2009). Thus, the neural substrates of imagination and invention enable the brain to formulate and construct tangible objects that provide future survival advantages to the individual, tribe or country. Examples include weapons, farming equipment and procedures, transportation vehicles (from horses to airplanes), drugs and medical devices, and sophisticated high-speed communication networks.

**Figure 3:**
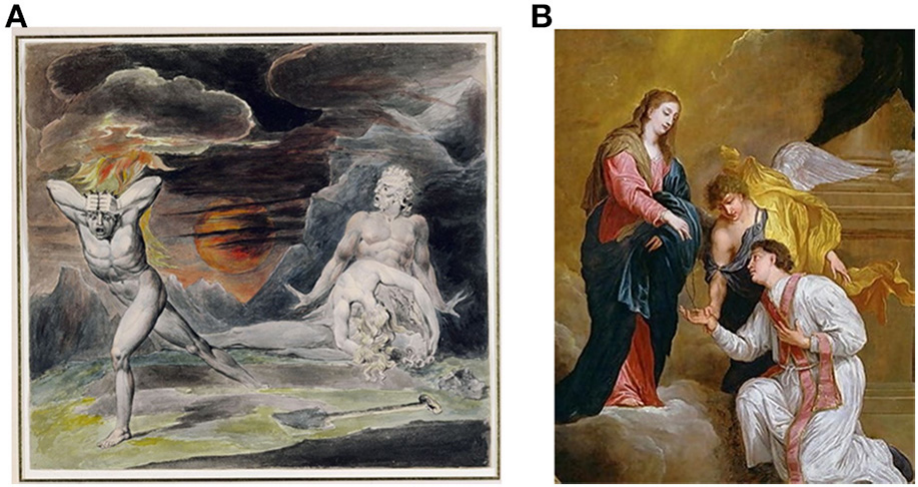
**Prominent historical examples of magical thinking, the belief in imaginary agencies, that became commonplace throughout the world, presumably because of its adaptive value for the individual and societies. (A)** “Cain Fleeing from the Wrath of God” William Blake, British (1757–1827). The belief that one will be punished by an imagined “God” for immoral acts would be expected to reduce the incidence of such acts. Examples include behaviors that adversely affect monogamous relationships (adultery) or violent acts against other members of a society (murder). **(B)** “Saint valentine receives a rosary from the virgin” David Teniers III, Flemish, (1600s). The belief in a loving God and “angels” can facilitate cooperation and caring for others.

A fascinating aspect of human SPP is the ability to fabricate mental entities that do not exist in the real world, including magical thinking. Magical thinking can be defined as “beliefs that defy culturally accepted laws of causality. In Western culture magical thinking refers to beliefs in, among other things, clairvoyance, astrology, spirit influences, and telepathy.” (Einstein and Menzies, 2004). Superstitions and rituals are examples of types of magical thinking. The cognitive fabrication of imaginary patterns is prominently illustrated in religious beliefs which have presumably provided an adaptive advantage to many societies. Magical thinking is at the core of all major religions wherein specific life events are believed to be controlled by “God,” and the “believers” behavior is designed to please “God” and avoid “his” wrath (Bloom, 2012). Figure 3 illustrates how a type of SPP, magical thinking, has had a major influence on cultural evolution. A recent functional MRI study suggests that religious belief involves neural networks that process information regarding intent and emotion, abstract semantics and imagery (Kapogiannis et al., 2009a). Transcranial magnetic stimulation focused on the left lateral temporal lobe, but not the right lateral temporal lobe or vertex, reduced magical thinking (Bell et al., 2007) providing further insight into the neural networks involved in magical thinking. Interestingly, structural differences between religious and non-religious subjects have been demonstrated including increased volume of right middle temporal cortex and reduced volumes of left precuneus and orbitofrontal cortex in religious subjects (Kapogiannis et al., 2009b). These findings are consistent with psychological theories of the evolution of religious belief which posit adaptive cognitive functions of such magical thinking (Culotta, 2009).

The history of “Gods” is that they are fabricated in the mind as explanations for phenomena that are not understood. Once those phenomena were explained by scientific investigation, those “Gods” were abandoned in due course. Take my Norse ancestors, for example, who fabricated Thor the “Thunder God” as an explanation for thunder storms. Nowadays most everyone accepts the fact that thunder storms are not caused by a “God,” but instead result from the rapid upward movement of moist warm air. In more recent history, the Gods of the major religions of the world (Christianity, Islam, Judaism..) are imagined to be “personal Gods” who can “answer prayers” heal the sick, and grant “eternal life” to those who follow “their” decrees. Of course the sacred texts that guide members of these religions were written by humans and contain fabricated tales of “creation,” “miracles” and “wrath” of God. Magical thinking may have resulted in the belief by many religions that humans are fundamentally different from other organisms, invoking their possession of a “soul” apart from the brain itself and believing themselves “created in God's image.” However, advances in knowledge of the molecular and cellular underpinnings of brain function during the past 50 years strongly suggest that Gods and an afterlife are delusions fabricated within and confined to neural networks of the human brain (Dawkins, 2006). Nevertheless, many dogmatically religious people continue to ignore the incontrovertible evidence for human evolution, and instead embrace the mythology of creation by a God (Coyne, 2012). This is a fascinating example in which a type of uniquely human SPP (magical thinking) has sustained a belief that is based on fantasy rather than reality.

Interestingly, as described in the next section, there is fine line between the magical thinking of religious beliefs that have provided adaptive value for many civilizations and societies, and the delusions of psychoses that can place individuals and those they associate with in harm's way. This notion is illustrated in the following two quotes which exhibit the remarkable similarities between the delusions of individuals with schizophrenia and those described in religious doctrine. “Because you think what you're believing is true,” she replied. “I used to know, beyond a shadow of a doubt, that—what other people would think was crazy—I thought was absolutely true, that I was privy to a special truth.” *Elyn Saks, Professor and schizophrenia patient*. “You have searched me, Lord, and you know me. You know when I sit and when I rise; you perceive my thoughts from afar. You discern my going out and my lying down; you are familiar with all my ways.” *Bible NIV, Psalm 139, verses 1-3*.

An understanding of the neurochemistry of delusions has emerged, in part, from studies of psychedelic drugs such as mescaline, LSD and psilocybin which often elicit mystical/spiritual experiences and magical thinking (Griffiths et al., 2011; Lyvers and Meester, 2012). Research on these drugs has revealed aspects of SPP in the human brain that are shared with lower mammals. First, the serotonin 5-HT(2A) receptor, which is conserved among mammals, is believed to be the molecular target of all three psychedelic drugs. Second, rodents exhibit behavioral responses to the psychedelic drugs consistent with altered perceptions/PP similar to humans including reduced impulsivity and altered time perception (Hanks and González-Maeso, 2013). Of course many people come to believe in imaginary agents/Gods (Christians and Muslims) or the “feeling of oneness” (Buddhism) not by taking psychedelics, but instead by indoctrination at an early age, with regular reinforcement with prayer and meditation. Interestingly, differences in serotonin receptor ligand binding between those who are and are not self-transcendent/religious have been reported (Borg et al., 2003). It will be of considerable interest to elucidate the SPP mechanisms (neural circuits and neurochemistry) that mediate reality-based thinking and magical thinking, and the transitions between these mental states.

## Aberrant pattern processing in psychiatric and degenerative brain disorders

The burden of brain disorders on societies is immense. Anxiety disorders and depression hobble the lives of hundreds of millions of people throughout the world (Baxter et al., 2014). Although effective treatments are available for many people affected with these disorders, many others continue to suffer. Less common, but more refractory to successful treatment, are bipolar disorder and schizophrenia (Saarni et al., 2010). Because psychiatric disorders often have an early age of onset (second, third and fourth decades of life) and can exhibit a relapsing and remitting course, their lifetime burden is substantial. Later in life many people develop a neurodegenerative disorder, with Alzheimer's disease (AD) and Parkinson's disease (PD) being the most prevalent (Chen, 2010; Reitz and Mayeux, 2014). AD and PD are progressive fatal disorders with the patients requiring constant care for 5–10 years. Here I consider the roles of aberrant and/or impaired PP in the pathogenesis of these neurological disorders.

In general, psychiatric disorders result from an abnormal skewing of SPP in ways that dissolve the neural circuit-based boundaries between reality and imagination, between the realms of possibilities and probabilities. There are likely evolution-based reasons that anxiety and depression, and “paranoia spectrum disorders” are so common. Everyone experiences anxiety transiently in situations that involve real threats to oneself or loved ones; this heightened state of arousal is an adaptive response that provides motivation toward actions that can mitigate the danger. However, individuals with an anxiety disorder react to perceived threats that either do not in fact exist or are highly unlikely to occur. Depression is a state of self-doubt and hopelessness that often follows a period of chronic anxiety or a catastrophic life event. It involves a pervasive distortion of reality and an unrealistic catastrophic view of the future. During their daily experiences individuals with chronic anxiety and depression perseverate on patterns of “worst case scenarios.” For example, a minor reprimand for an error made at work may be mentally magnified into a pervasive rumination on a scenario in which the person is fired and can no longer support his/her family, etc. Central to the perturbed thought processes in these disorders is the complex SPP repertoire of humans including language and imagination. The cellular and molecular alterations underlying anxiety disorders and depression are partially understood, and involve reductions in synaptic densities in the hippocampus, deficits in serotonergic and noradrenergic neurotransmission, and reduced BDNF signaling (McEwen et al., 2012; Blier and El Mansari, 2013). Accordingly, treatments that stimulate BDNF signaling, including exercise, serotonin/norepinephrine reuptake inhibitors and electroconvulsive shock therapy are effective in many patients (Domingos da Silveira da Luz et al., 2013; Duric and Duman, 2013). Studies of animal models have clearly shown that BDNF plays a major role in spatial pattern separation, and that interventions known to elevated BDNF levels and ameliorate anxiety and depression enhance spatial pattern separation (Creer et al., 2010; Bekinschtein et al., 2013). These findings suggest roles for impaired PP in the pathogenesis of anxiety disorders and depression.

Based upon reciprocal social interactions, language-based communication and the perception of others' emotions, in light of their own introspective thoughts and emotions, humans presume that others have a mind similar to their own. By envisioning future interactions with other individuals, the outcomes of those future interactions can be biased in one's favor. Similarly, the ability to predict the thought processes and actions of others provides major advantages in navigating what are often complex social landscapes in human societies. However, the inferring of agency, beliefs and intentions in others, is dysregulated in schizophrenia, bipolar disorder and autism spectrum disorders (Senju, 2012; Martin et al., 2014). The diagnosis of schizophrenia is based on a person suffering from delusions, hallucinations and disorganized speech, often accompanied by or oscillating with negative symptoms such as anhedonia, social isolation, and lack of motivation. A key feature of schizophrenia is a blurring of the lines between external reality and internal imagination, between patterns that are real and those that are mentally fabricated (Larøi et al., 2012; Howes and Murray, 2014). The hallucinations and paranoia that occur schizophrenia patients could be considered a pathological dysregulation of the imagination and mental time travel categories of SPP.

In schizophrenia, patterns generated within the brain are perceived as external. At other times, schizophrenia patients may experience paranoia, an unrealistic belief that others intend to harm them, and a fear of persecution. Such delusions can severely compromise the ability of schizophrenics to perform well in school, a job and social settings. However, more subtle paranoid beliefs are common among the general population, and may often be beneficial. For example, when returning to one's car in a dark parking lot, the belief that there may be a robber lurking in the vicinity will prompt behaviors that reduce the chance of being mugged. Similarly, not providing personal information to strangers can prevent identity theft. For most high-functioning individuals in modern society there is a balance between trust and paranoia, based upon a rationale appraisal of the situation based upon experience and understanding (Green and Phillips, 2004; Nelson et al., 2012). It has been proposed that religion and schizophrenia have similar evolutionary underpinnings (Dein and Littlewood, 2011). In this view, both theory of mind and attributing life events to an external agency are hyperextended and intrude thought processes and actions excessively. One way to view the brains of those who believe in a God, particularly fundamentalists, is that they have a low level of trust in people and so fabricate an external agency, a security blanket in which they trust.

It can be argued that although their symptoms are different, human psychiatric disorders all involve distortions of reality (hallucinations in schizophrenia; unrealistic view of threats to well-being in anxiety and obsessive-compulsive disorders; and a distorted image of one's current and future life situations in depression). In essence, the SPP capabilities of imagination and envisioning future scenarios are dysregulated. Interestingly, structural and/or neurochemical abnormalities in the prefrontal cortex have been implicated in each of the major psychiatric disorders (Huey et al., 2008; Rigucci et al., 2010; Teffer and Semendeferi, 2012), again highlighting the importance of the evolved human prefrontal cortex in both normal and pathological aspects of SPP.

**Figure 4:**
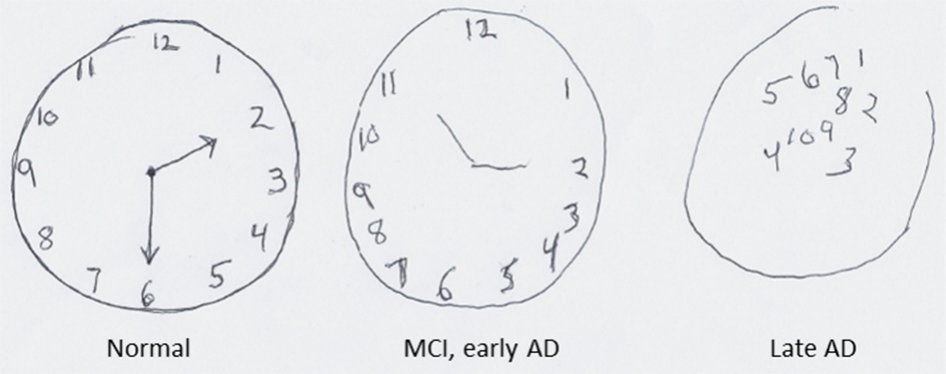
**The progressive deterioration of pattern processing ability in a subject as they progress from mild cognitive impairment (MCI) to severe Alzheimer's disease (AD)**. In this clock drawing task the subject is asked to draw a clock with the hours and showing the time 2:30. When the person has MCI/early AD the numbers for the hours on the clock are drawn in proper order, but during the time it took to draw the clock the subject forgot that he/she had been asked to show the time 2:30. In the case of the patient with late-stage AD, the drawing bears little resemblance to a clock.

Alzheimer's disease (AD) is rapidly becoming a major cause of morbidity and mortality as an increasing number of people live into their 70 and 80 s, the most common age of disease onset (Mayeux and Stern, 2012; Chan et al., 2013). AD is characterized by the progressive deterioration of cognitive function with an insidious impairment of short-term memory that becomes amplified to the point of inability to perform even the simplest tasks. As synapses and neurons degenerate in the hippocampus and connected regions of cerebral cortex PP ability is lost. One of the cognitive tests routinely used in the diagnosis and evaluation of disease progression in AD is the clock drawing test in which the subject is asked to draw a clock that includes the hour numbers 1–12 and shows a specific time. Figure 4 shows examples of a clock drawing test when a subject progresses from normal brain function to mild cognitive impairment (MCI) or early AD to advanced AD. This example of progressive deterioration of clock drawing ability in AD illustrates how the degeneration of brain regions involved in SPP can manifest as an all too common human brain disorder. Aging is the major risk factor for AD. During normal aging there is often a decrement in spatial pattern separation performance (Stark et al., 2010) which may contribute to age-related episodic memory deficits (Holden and Gilbert, 2012). Many individuals may compensate for such age-related alterations, and some cases of AD may result from a failure of such adaptive neuroplasticity (Buckner, 2004).

## Predictions of the SPP theory of human brain evolution

If SPP has played a fundamental role in the evolution of the human brain, then this should be evident in both the historical record and trajectories of different human populations throughout the world. The SPP theory predicts that populations that more rapidly develop SPP capabilities will experience accelerated accrual of resources and prosperity. The examples of major SPP abilities acquired during human evolution that were considered above (language, invention, imagination, reasoning, and planning for the future) should have each provided a survival and resource-accumulating advantage. The SPP theory therefore predicts that populations that did not develop each of these SPP capabilities would have been outcompeted by those populations with brains that did acquire, through evolution, those SPP capabilities. This prediction is supported by the fact that all surviving populations of *H. sapiens* use language, invent tools and exhibit imagination and complex reasoning. Hominin populations lacking, or with relatively poorer, SPP capabilities presumably failed to compete successfully, and so no longer exist.

The numbers of extinct hominin species is uncertain, estimates range from 8 to 27, with evidence suggesting that Neanderthal and Denisova hominins extinctions were driven by *H. Sapiens*, the only surviving species (Bokma et al., 2012). The pattern processing capabilities of our most recent ancestral species cannot be fully established, but did include the invention/use of simple tools, drawing skills, and some forms of gesture-based and verbal communications. One prediction of the SPP hypothesis is that the development of qualitatively and/or quantitatively new pattern processing capabilities conferred a survival advantage to *H. Sapiens*. Brain size deduced from cranial vault volume varies among now extinct hominins, with most considerably smaller than humans and some, notably Neanderthals, close to *H. Sapiens* (Arsuaga et al., 2014). As described above the expansion of the prefrontal cortex and brain regions associated with processing of visual input in humans relative to great apes, suggests that the functional roles of these brain regions likely provide major adaptive advantages. A prediction of the SPP theory is that there was a progressive increase in the size and synaptic/functional capabilities of these brain regions as hominin species evolved. Unfortunately, the latter prediction is not directly testable given the lack of brain specimens from any extinct hominin species.

The SPP theory predicts that variability in SPP capabilities among current human populations will be associated with variations in resources, health and welfare (indicators of fitness) of the different populations. Studies have documented positive associations of brain size with greater intelligence, faster decision making and greater cultural achievements between and within genetically differentiated populations of modern humans (Rushton and Jensen, 2008). This suggests that variability in SPP among existing groups of humans may be sufficiently robust to influence their relative fitness and so the future evolution of the human brain. The differential SPP-mediated development of technologies to improve transportation, manufacturing, scientific discovery and health care have resulted in the advancement of some populations above others. Individuals in populations that have most heavily utilized the SPP capabilities of their brains currently enjoy the greatest levels of prosperity, better health and longer lives. The disparities between and within countries are in some cases quite striking, with African countries exhibiting considerably less propensity for SPP, as reflected in poverty, low levels of education, high infant mortality and short lifespans. In contrast, the United States, and many countries in Europe and Asia are experiencing economic growth that is arguably resulting, in large part, from development of SPP-based technologies, with computer-based systems being a prominent example of a human invention that enables processing of information at rates many orders of magnitude beyond the capability of the human brain. Clearly, humans have recognized the central importance of SPP for their advancement as a species.

Interestingly, although variability in DNA sequences is the fundamental molecular basis of evolution, emerging evidence suggests major roles for trans-generational epigenetic mechanisms for the rapid inheritance of traits. For example, changes in DNA methylation can occur in response to life experiences, and such methylation changes can affect gene expression and phenotypes influenced by those genes. The brain may be particularly modifiable by epigenetic processes that can influence its development, function and susceptibility to behavioral disorders (Meloni, 2014). Animal studies have shown that changes in DNA methylation that occur in brain cells in response to socio-environmental factors (particularly during early life) play important roles in learning and memory and, importantly, can be transferred from parents to offspring. This has been shown for several behavioral traits in animals including sociality, and susceptibility to addiction or depression (Meloni, 2014; Sen, 2014; Vassoler and Sadri-Vakili, 2014). If epigenetic mechanisms influence SPP, then it would be predicted that SPP capabilities may be rapidly enhanced within only a few generations.

Finally, the SPP theory predicts that human evolution will continue to involve expansion of the prefrontal cortex and functionally associated brain regions, with resulting improvements in the brain's ability to rapidly process information and make (good) decisions. The specific outcomes of advanced SPP for future generations remain to be determined, but may (hopefully) include the invention of technologies that eliminate suffering and help ensure the long-term survival of our species.

## Societal implications

This closing section is intended to highlight the importance for society of advancing an understanding of how and why SPP is fundamental to the human experience. To illustrate this point, examples are provided of how SPP has enabled the development of major human cultural practices that are not based in reality.

Considerable evidence, some of which is described above, supports the notion that SPP is the underlying mechanism for many higher cognitive functions of humans (thought, reasoning, imagination, invention). This suggests that humans are the dominant species, in part, because of the superior ability of their brains to store and process patterns and transfer those patterns to others. Technologies developed by the human brain are based on the reproduction and modification of patterns encountered in nature (e.g., airplanes designed around birds), and/or fabrication of new patterns (e.g., the computer I am using to write this article). While the centrality of SPP to all of human consciousness, thoughts and behaviors is congruent with our evolutionary history, some neuroscientists and most of the lay public are unaware of this important fact. For example, in an interesting discourse on how “our minds make inferences that appear to go far beyond the data available,” Tenenbaum et al. (2011) do not consider advanced PP as the answer to this question and, instead, conclude that “Deeper is a framework for understanding why the mind works the way it does, in terms of rational inference adapted to the structure of real-world environments, and what the mind knows about the world, in terms of abstract schemas and intuitive theories revealed only indirectly through how they constrain generalizations.” For the reasons described in the preceding sections, SPP mediated by brain regions expanded in humans (PFC, in particular) may underlie the ability of humans to make intuitive and rational inferences.

Knowledge of the roles of SPP in human brain function could be incorporated into the primary education so that everyone has a basic understanding of the mechanisms underlying human cognition, creativity and complex behaviors. The ability to distinguish reality from fantasy is one example where education is lacking such that a considerable portion of the human population is unaware of the fact that supernatural entities such as Gods are fabricated within the human brain, and that belief in such mentally fabricated entities is learned from other “believers.” While the percentage of Americans who believe in God is slowly declining, and those that believe in evolution is increasing, recent polls indicate that approximately 75% continue to believe in the actual existence of a “God” or “universal spirit” (Galanter, 2010; Harris Poll, 2013^1^). Indeed, many individuals in positions of authority espouse an external “God” as their guiding “force.” For example, a recent President of the United States stated in a public speech that “I am driven with a mission from God. God would tell me, ‘George go and fight these terrorists in Afghanistan.’ And I did. And then God would tell me ‘George, go and end the tyranny in Iraq.’ And I did.” George W. Bush. August, 2003. *Sharm el-Sheikh*. This type of delusional SPP has been a justification for violence for millennia and, indeed, is the justification used by the Islamic extremists who chanted “Allâhu Akbar” (God is great) as they crashed airplanes into the World Trade Center buildings on September 11, 2001 (McTernan, 2003). Another example comes from the geneticist and current director of the National Institutes of Health who wrote: “God's domain is in the spiritual world, a realm not possible to explore with the tools and language of science. It must be examined with the heart, the mind, and the soul—and the mind must find a way to embrace both realms.” Francis S. Collins, *The Language of God* (2006). On the contrary, from the perspectives of neuroscience, realism and moralism, it is important for the advancement of modern societies that children not continue to be deceived into “embracing both realms,” and instead be taught to distinguish reality from fantasy, and to embrace reality and enjoy fantasy. The mental fabrication of “spiritual worlds” can certainly be comforting, and the pursuit of valuable aspects of the major religions such as morality and the principles of “oneness” and caring for one another should continue, albeit with the knowledge of their evolutionarily conserved neural substrates.

Compared to lower species, the human brain is particularly advanced in its ability to fabricate new patterns and transfer them to others. This SPP ability has been fundamental to the development of new technologies and to the dissemination of knowledge of the world and its societies. What are the implications of SPP being the neural basis for the entire human experience for the future of human societies? Computers can now perform many types of pattern processing and are increasingly used to replace people in positions such as accounting, data processing and manufacturing. While computers still fall considerably short of humans in the realms of invention and scientific discovery, one might imagine that as understanding of the mechanisms by which neural circuits in the human brain process patterns increases, computers and robots may equal or even surpass humans in the areas of creativity, invention and even scientific discovery (Sparkes et al., 2010). Artificial intelligence is an active area of investigation involving efforts to mimic the brain's SPP capabilities on the one hand, and to interface the brain with machines, on the other hand (Meltzoff et al., 2009; Fingelkurts et al., 2012). A better understanding of SPP mechanisms, at the molecular, cellular, neuronal network and behavioral levels will not only advance knowledge of brain function and neurological disorders, but may also inform research in wide range of fields of technology.

### Conflict of interest statement

The author declares that the research was conducted in the absence of any commercial or financial relationships that could be construed as a potential conflict of interest.

## Footnotes

^1^http://www.harrisinteractive.com/NewsRoom/HarrisPolls/tabid/447/ctl/ReadCustom%20Default/mid/1508/ArticleId/1353/Default.aspx
